# Case Report: Post-transplant lymphoproliferative disorder after kidney transplantation in a child with schimke immuno-osseous dysplasia

**DOI:** 10.3389/fimmu.2026.1740720

**Published:** 2026-01-15

**Authors:** Peng Liu, Xiyan Tian, Daokui Ding, Lei Wang, Peile Wang

**Affiliations:** 1 Department of Pediatric Intensive Care Unit, the First Affiliated Hospital of Zhengzhou University, Zhengzhou, China; 2 Department of Pediatric Surgery, the First Affiliated Hospital of Zhengzhou University, Zhengzhou, China; 3 Department of Pathology, the First Affiliated Hospital of Zhengzhou University, Zhengzhou, China; 4 Department of Pharmacy, the First Affiliated Hospital of Zhengzhou University, Zhengzhou, China

**Keywords:** epstein-barr virus, inborn error of immunity, kidney transplantation, post-transplant lymphoproliferative disorder, schimke immunoosseous dysplasia

## Abstract

With the inherent T-cell immunodeficiency of Schimke immune-osseous dysplasia (SIOD), the management of immunosuppressive therapy after transplantation and life-threatening infections remains a challenge. Here, we present a case of a child with SIOD who developed early-onset Epstein-Barr virus (EBV)-associated post-transplant lymphoproliferative disorder (PTLD) after kidney transplantation. PTLD frequently involves the gastrointestinal tract and solid allografts, while this case also involved the lungs, which is extremely rare. This case underscores the importance of considering PTLD in recipients with immunodeficiency, long-term immunosuppressive therapy, and EBV seronegativity. It also suggests low-dose immunotherapy and hematopoietic stem-cell transplantation for patients with SIOD.

## Introduction

Schimke immuno-osseous dysplasia (SIOD), first described by Schimke et al. in 1971, is a rare autosomal recessive multisystem disorder with an incidence of 1 per 1–3 million live births (1, 2). The condition is caused by biallelic pathogenic variants in the *SMARCAL1* gene, which encodes a protein belonging to the SWI/SNF protein family in chromatin remodeling and the transcriptional regulation of certain genes (3). Characteristic clinical manifestations include short stature, spondyloepiphyseal dysplasia, steroid-resistant nephrotic syndrome, and T-cell immunodeficiency (4). This immunodeficiency, present in approximately 80% of individuals with SIOD, is associated with a lack of interleukin-7 (IL-7) receptor alpha expression on T cells and their poor response to recombinant IL-7 (5). The impaired T-cell signaling in SIOD is not due to an intrinsic defect but results from diminished hematopoietic precursor proliferation and compromised T-cell function (6). This view is supported by studies on *SMARCAL1* knockout mice, which have found increased DNA damage and apoptosis in hematopoietic stem/progenitor cells and developing thymocytes (7). In addition, mature T cells in SIOD exhibit a predominantly proinflammatory Th1 skew with signs of exhaustion (8). For patients with SIOD, infection associated with T-cell deficiency is generally the most common complication and a major cause of mortality (2, 9). A 20-year cohort study showed the five-year overall survival rate was 89%, and the ten-year rate was 10% (10).

SIOD varies in severity, ranging from prenatal growth deficiency in the first few years of life to slow progression to end-stage renal disease, ultimately necessitating renal dialysis and/or renal transplantation (11). However, for those who undergo kidney transplantation, the inborn error of immunity of SIOD combined with post-transplant immunosuppression management often leads to recurrent severe infections caused by bacteria, viruses, and fungi (12, 13).

Post-transplantation lymphoproliferative disorder (PTLD), a serious and often devastating malignant complication, is closely associated with Epstein-Barr virus (EBV). PTLD incidence follows a bimodal curve, with an initial spike during the first year and a second spike typically occurring 5–15 years after transplantation. The disease frequently presents with extranodal involvement, most commonly affecting the gastrointestinal tract, solid allografts, and the central nervous system (14).

Herein, we present a case of early-onset, EBV-associated polymorphic PTLD involving the gastrointestinal tract, lymph nodes, and lungs in a child with SIOD following kidney transplantation.

## Case presentation

**Figure 1:**
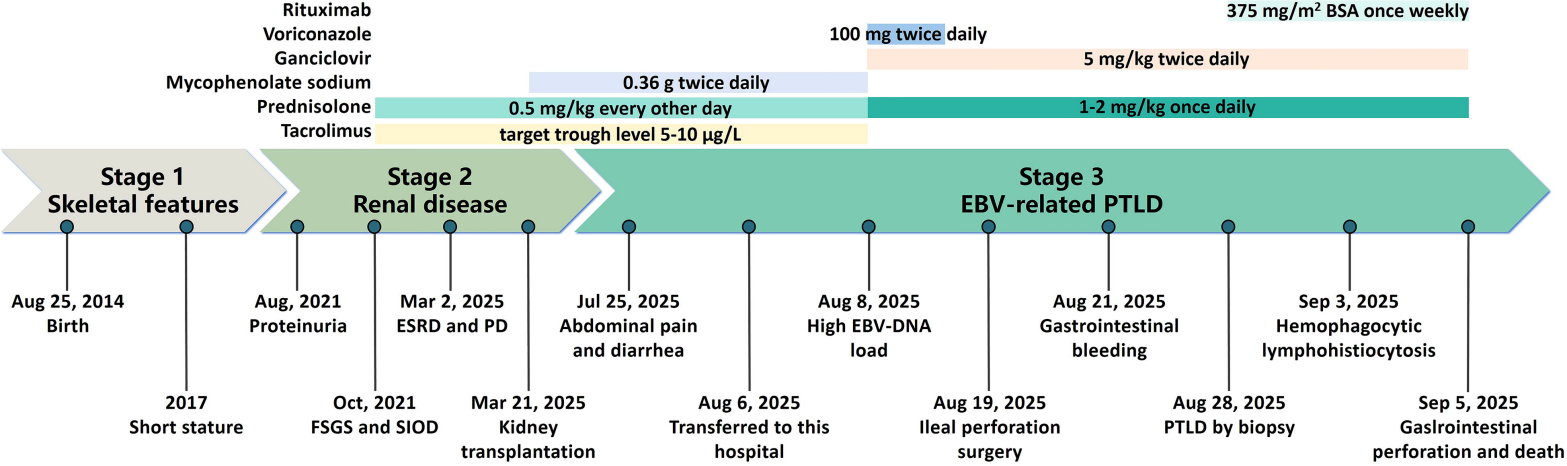
The detailed diagnosis and treatment process of the child. FSGS, focal segmental glomerulosclerosis; SIOD, Schimke immuno-osseous dysplasia; ESRD, end-stage renal disease; PD, peritoneal dialysis; EBV, Epstein-Barr virus; PTLD, post-transplant lymphoproliferative disorder; BSA, body surface area.

An 11-year-old boy was transferred to this hospital on 6 August 2025, presenting with an 11-day history of aggressive abdominal pain, diarrhea, and intermittent fever (**Figure 1**). He was the second child of a non-consanguineous marriage. His parents and brother were healthy and had normal stature. He was born at 38 + 2 weeks’ gestation with a 2.1-kg birth weight.

The medical history was summarized in **Table 1**. The patient first presented with proteinuria and short stature in 2021 (**Table 1**). A subsequent renal biopsy revealed focal segmental glomerulosclerosis (FSGS). Immunosuppressive therapy with tacrolimus (target trough level 5-10 μg/L) and low-dose prednisolone (0.5 mg/kg every other day) was initiated. Exome sequencing identified compound heterozygosity of a paternal splicing donor mutation c.1334 + 1 G>A (rs1384402545) with a minor allele frequency of 0 in the Genome Aggregation Database (gnomAD), and a maternal nonsense mutation c.1051 C>T (p. Gln351Ter, rs2469621143) with a minor allele frequency of 0 in gnomAD in the *SMARCAL1* gene. According to the American College of Medical Genetics and Genomics (ACMG) guidelines, the variant c.1334 + 1 G>A was classified as likely pathogenic and c.1051 C>T as pathogenic, confirming a diagnosis of SIOD. Over the following four years, the patient experienced intermittent neutropenia and anemia, and underwent recurrent upper respiratory infections, bronchitis, and mild pneumonia (at least 5–6 times annually). By March 2025, he had progressed to end-stage renal disease (ESRD) and underwent allogenic kidney transplantation after three weeks of peritoneal dialysis. Pre-transplant laboratory tests showed cytomegalovirus seropositivity (<500 copies/mL), EBV seronegativity (<500 copies/mL, EBV seropositivity for the donor), and lymphopenia (**Table 1**). After kidney transplantation, mycophenolate sodium (0.36 g twice daily) was added.

**Table 1:** Laboratory data across multiple time points.

Parameter	At the time of SIOD diagnosis	Before kidney transplantation	At the time of PTLD diagnosis	Normal values
Height (cm)	108	120	120	–
Weight (kg)	17.5	21	15.6	–
Body-mass index (kg/cm^2^)	15.0	14.6	10.8	18.5-24.9
Leukocyte (10^9^/L)	8.4	3.95	1.06	3.5-9.5
Erythrocyte (10^12^/L)	5.18	2.74	3.15	4.3-5.8
Hemoglobin (g/L)	154	85	112	130-175
Platelets (10^9^/L)	241	121	43	125-350
Neutrophil (10^9^/L)	6.39	1.25	0.39	1.8-6.3
Creatinine (μmol/L)	31	689	77	20-115
Total bilirubin (μmol/L)	1.10	2.70	4.5	0-25
Lactate dehydrogenase (U/L)	316	246	465	100-300
IgG (g/L)	6.86	6.94	5.32	5.66-14.25
CD45+ lymphocytes (/μL)	1636	514.66	335.81	1530-3700
CD3+ T cells (/μL)	619	302.66	275.21	770-2860
CD4+ T cells (/μL)	235	232.24	23.59	414-1440
CD8+ T cells (/μL)	372	66.19	243.33	238-1250
CD19+ B cells (/μL)	794	89.96	6.87	90-560
Tacrolimus trough concentration (μg/L)	8.7	5.4	7.0	5-10

SIOD, Schimke immuno-osseous dysplasia; PTLD, post-transplant lymphoproliferative disorder.

**Figure 2:**
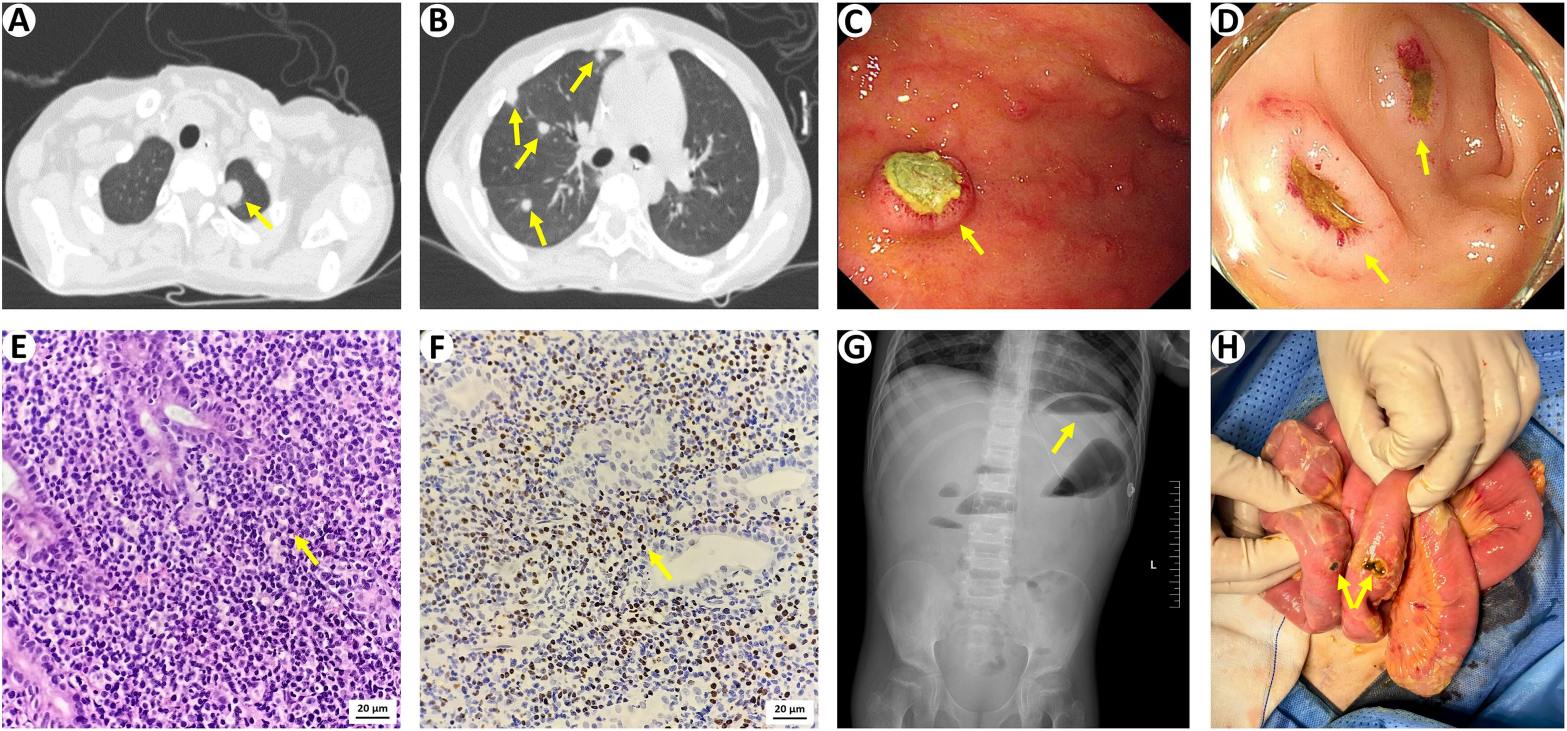
Diagnosing EBV-associated post-transplant lymphoproliferative disorder. **(A, B)** Chest CT scans showing solid nodules diffused in bilateral lungs (arrows). **(C, D)** Gastrointestinal endoscopic images showing multiple mucosal bulges with ulcers in the stomach and sigmoid colon (arrows). **(E)** Histology of gastric biopsies showing a dense polymorphous lymphoplasmacytic infiltration (arrow; haematoxylin and eosin staining, 40×10 magnification). **(F)** Chromogenic *in-situ* hybridization for EBV-encoded RNA is positive within neoplastic lymphocyte nuclei (arrow; brown signals, 40×10 magnification). **(G)** Abdominal X-ray scans showing free air beneath the left diaphragm. **(H)** Intraoperative image demonstration of ileal perforation. EBV, Epstein-Barr virus.

On physical examination, the patient was short-statured, malnourished, and had lost 2 kg in the past 4 weeks. Laboratory tests showed high EBV-DNA load (8990 copies/mL, reference range <500 copies/mL), elevated inflammatory markers (C-reactive protein 23.04 mg/L and procalcitonin 0.81 ng/mL), pancytopenia, and lymphopenia (**Table 1**). Chest computed tomography (CT) revealed solid nodules diffused in bilateral lungs (**Figures 2A, B**). Metagenomic next-generation sequencing (mNGS) of bronchoalveolar lavage fluid (BALF) detected EBV with 692746 reads. Gastrointestinal endoscopy revealed multiple mucosal bulges with ulcers scattered in the stomach, duodenal bulb, and entire colon (**Figures 2C, D**). Histopathological examination of the gastric and sigmoid colon samples identified that most lymphocytes were positive for EBV-encoded RNA via chromogenic *in-situ* hybridization, suggesting polymorphic lymphoproliferative disease (**Figures 2E, F**). These led to a diagnosis of early-onset EBV-associated polymorphic PTLD.

Considering the high EBV-DNA load, tacrolimus and mycophenolate sodium were discontinued. The patient was started on intravenous methylprednisolone (1–2 mg/kg once daily) and ganciclovir (5 mg/kg twice daily). After the diagnosis of PTLD, rituximab (375 mg/m^2^ body surface area once weekly) was added. On day 13 of admission, he underwent emergency laparotomy for ileal perforation (**Figures 2G, H**). Histopathological results of mesenteric lymph nodes confirmed the diagnosis of PTLD. Subsequently, his course was complicated by persistent gastrointestinal bleeding on day 16 and hemophagocytic lymphohistiocytosis on day 29, which was evidenced by fever (> 38 °C), hyperferritinemia (ferritin 24196 μg/L; reference range 30-400 µg/L), hypertriglyceridemia (triglycerides 7.83 mmol/L; reference range <1.7 mmol/L), and pancytopenia. Unfortunately, the patient died 31 days after admission because of perforation recurrence.

## Discussion

PTLD is a well-known malignancy complication in children after transplant, with a high mortality of 30-70% (15). To date, established risk factors for developing early-onset PTLD include the type of transplanted organ (intestinal > lung, liver, heart > kidney), EBV mismatch at time of transplantation (D+/R−), intensity of induction immunosuppressive therapy and duration of maintenance therapy (including graft rejection episodes), polyclonal anti-lymphocyte antibodies, young age, and non-Hispanic white race/ethnicity (14, 16). This patient was 11 years old, EBV seronegative, and had a history of long-term immunosuppressive therapy, all of which indicated he was at high risk of PTLD.

The clinical presentation of PTLD can be either nonspecific (fever, weight loss, allograft dysfunction, or anemia) or reflect the site of localization of the mass. It frequently involves the gastrointestinal tract, lymph nodes, solid allografts, and, rarely, the lungs (17). In addition to the lymph node and gastrointestinal symptoms, rare solid nodules in both lungs were observed in this case. Empirical voriconazole (100 mg twice daily) was added but discontinued after 7 days due to enlarged pulmonary nodules and the absence of respiratory symptoms. Without a lung biopsy and positron emission tomography-CT (PET-CT), PTLD involving the lungs was considered because of positive mNGS results in BALF.

Therapeutic strategies for PTLD include reducing immunosuppression, surgery or radiation, rituximab monotherapy, chemotherapy, and antiviral therapy. The cornerstone of the initial management for PTLD is to reduce immunosuppression to partially restore EBV-specific cellular immunity (14). In normal circumstances, EBV incorporates the normal B-cell program, promoting the expression of different latent antigens during B-cell development. These antigens elicit T-cell responses that destroy the majority of EBV-infected B cells. However, this immunologic response is diminished in transplant recipients, leading to B-cell transformation and PTLD (17). In this case, SIOD-related inborn error of immunity may further weaken the patient’s immune response. This may explain the rapid disease progression and poor prognosis, which occurred despite the immediate discontinuation of immunosuppressants upon diagnosis.

Nevertheless, the treatment in this case may need improvement. First, frequent monitoring of EBV load. In particular, for individuals at high risk of PTLD, closely observing for PTLD-related clinical manifestations (fever, diarrhea, lymphadenopathy, graft failure, etc.) is also necessary (14, 18). Second, adopt low-dose immunotherapy or immunosuppressive monotherapy after kidney transplantation. After transplantation, achieving a balance between immunosuppressive therapy and increased susceptibility to infections remains a challenge. Finsen et al. and Lücke et al. reported that six children successfully reduced immunosuppressive therapy to monotherapy with either MMF, rapamycin, or tacrolimus (3-5 μg/L) after kidney transplantation (12, 19). Therefore, SIOD patients can benefit from reduced immunosuppressants, and low-dose immunotherapy or monotherapy is recommended (20). Third, perform sequential hematopoietic stem cell transplantation (HSCT) and kidney transplantation. It has been reported that kidney transplantation followed by HSCT is a successful way to induce immune tolerance of allografts without immunosuppressants and correct the primary immunodeficiency of 3 children with SIOD (4). Given that the patient had neutropenia, anemia, and recurrent infections prior to kidney transplantation, HSCT might be a better option.

Due to the rarity and complexity of SIOD, there are currently no consensus clinical diagnostic and treatment criteria for the disease (4). The treatment tactics for SIOD primarily focus on symptomatic and supportive therapy, as well as delaying the progressive occurrence of kidney failure (21). Symptom relief and complete cure of SIOD primarily depend on kidney transplantation and HSCT. So far, a series of cases of children with SIOD who successfully underwent kidney transplantation have been published (2, 12, 22). However, patients were still at high risk of severe infections (13, 23, 24). Regarding HSCT, early cases reported by Baradaran-Heravi et al. found that 4 out of 5 patients died during the HSCT process or due to post-transplant complications (25). In contrast, Bertaina et al. recently described a successful sequential stem cell-kidney transplantation strategy using the same donor in 3 patients with SIOD, who achieved immune reconstitution and were able to discontinue immunosuppressants several months after kidney transplantation (4). Pehlivanoğlu et al. also successfully performed HSCT from the same donor on a child with SIOD who developed PTLD (26). Both studies adopted haploidentical HSCT-kidney transplantation from the same donor and reduced-intensity conditioning. Due to limited cases, the therapeutic efficacy and safety of kidney transplantation and HSCT still need further research.

This case has several limitations. First, the absence of lung biopsy or PET-CT precluded a definitive diagnosis of pulmonary nodules, which could only be inferred from the patient’s symptoms and EBV-positive in BALF. Second, testing for T cell function was not performed, which would have provided more reliable data for in-depth research into SIOD.

## Conclusion

For patients with SIOD, PTLD is a life-threatening complication of transplantation. This report presents a child with SIOD who developed PTLD involving the lungs after kidney transplantation. This case highlights the importance of considering PTLD in recipients with immunodeficiency, long-term immunosuppressive therapy, and EBV seronegativity. In addition, our experience suggests low-dose immunotherapy and HSCT for patients with SIOD.

## Data Availability

The original contributions presented in the study are included in the article/supplementary material. Further inquiries can be directed to the corresponding author.
